# Proteomic Analysis of Histone Variants and Their PTMs: Strategies and Pitfalls

**DOI:** 10.3390/proteomes6030029

**Published:** 2018-06-21

**Authors:** Sara El Kennani, Marion Crespo, Jérôme Govin, Delphine Pflieger

**Affiliations:** 1 University Grenoble Alpes, CEA, INSERM, BIG-BGE, 38000 Grenoble, France; marion.crespo@cea.fr (M.C.); jerome.govin@univ-grenoble-alpes.fr (J.G.); 2 CNRS FR 3425, BIG-BGE, F-38000 Grenoble, France

**Keywords:** histone variants, post-translational modifications, mass spectrometry, bottom-up analysis, computational tools, crosstalk

## Abstract

Epigenetic modifications contribute to the determination of cell fate and differentiation. The molecular mechanisms underlying histone variants and post-translational modifications (PTMs) have been studied in the contexts of development, differentiation, and disease. Antibody-based assays have classically been used to target PTMs, but these approaches fail to reveal combinatorial patterns of modifications. In addition, some histone variants are so similar to canonical histones that antibodies have difficulty distinguishing between these isoforms. Mass spectrometry (MS) has progressively developed as a powerful technology for the study of histone variants and their PTMs. Indeed, MS analyses highlighted exquisitely complex combinations of PTMs, suggesting “crosstalk” between them, and also revealed that PTM patterns are often variant-specific. Even though the sensitivity and acquisition speed of MS instruments have considerably increased alongside the development of computational tools for the study of multiple PTMs, it remains challenging to correctly describe the landscape of histone PTMs, and in particular to confidently assign modifications to specific amino acids. Here, we provide an inventory of MS-based strategies and of the pitfalls inherent to histone PTM and variant characterization, while stressing the complex interplay between PTMs and histone sequence variations. We will particularly illustrate the roles played by MS-based analyses in identifying and quantifying histone variants and modifications.

## 1. Introduction

The nucleosome core particle packaging cellular DNA is composed of two copies of H2A, H2B, H3, and H4 histones surrounded by 142 base pairs of DNA [1]. At the entry and exit sites on the surface of the nucleosome, DNA is bound by a linker histone H1 to stabilize the chromatin subunit structure [2,3,4]. Each histone comprises a globular domain, interacting with DNA and amino terminal tails protruding from nucleosome particles.

A large diversity of post-translational modifications (PTMs) has been widely described on histones. They include classic phosphorylation, acetylation, and methylation, but also acyl groups such as propionyl, butyryl, 2-hydroxyisobutyryl groups, and sugar derivatives (O-GlcNAcylation) [5]. They accumulate in the exposed surfaces of histones when incorporated into nucleosomes, namely their N- and C-terminal regions. However, several modifications have been identified within their globular domain (histone fold) and regulate their assembly into nucleosomes [6,7]. Histone PTMs are essential elements of chromatin signaling pathways. They accumulate at specific regions of the genome and participate in the definition of their functionality as active promoters, enhancers or in the maintenance in a robustly repressed status. As an example, the methylation of lysine 4 of H3 (H3K4) is a strong mark of active transcription. It is deposited by the methyltransferase Set1 from the COMPASS complex. These PTMs can recruit a variety of specific binders, which can either reinforce transcription activation or promote its repression (reviewed in Reference [8]). Another example is the methylation of H3K27 by Polycomb group proteins which establish a strongly repressed chromatin structure. Altogether, histone modifications are key features of chromatin dynamics and transcription regulation mechanisms with crucial roles during embryonic development and cell differentiation [9]. They have also been linked to a subset of diseases, including cancer, neurological, and autoimmune disorders [10].

**Figure 1:**
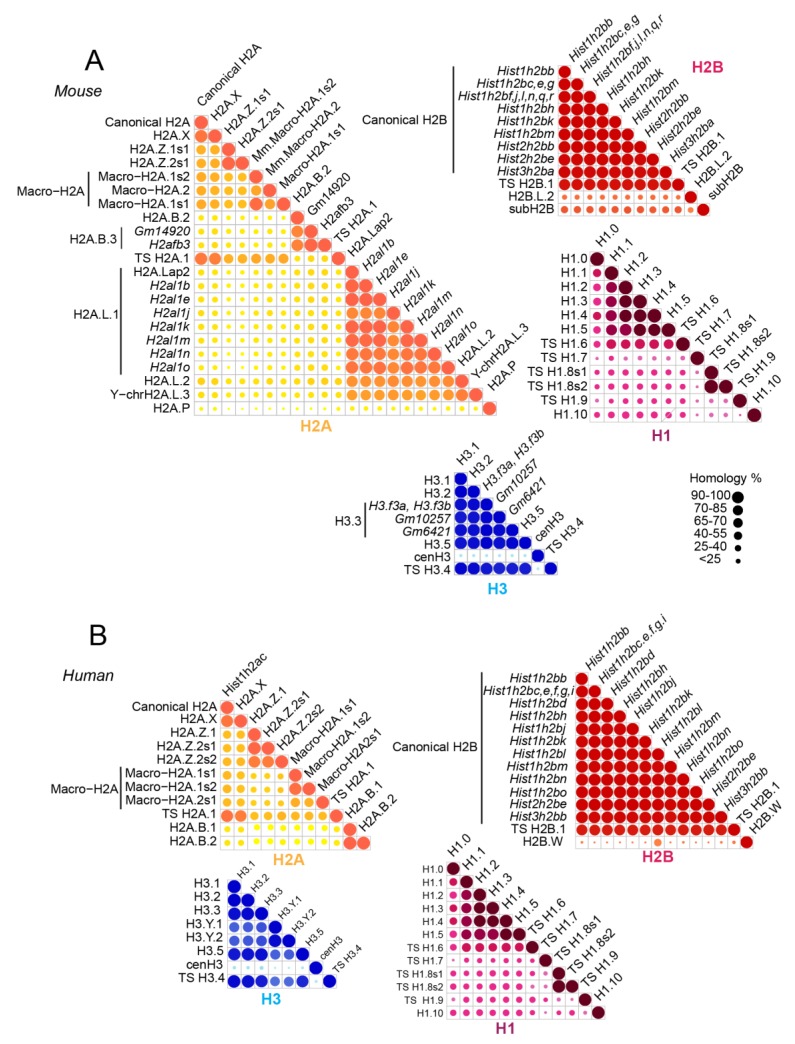
Sequence similarity between histone variants in human and mouse. Core histones H2A (yellow), H2B (red), H3 (blue), and histone linker H1 (purple) are illustrated for mouse (**A**) and human (**B**). Sequence data were obtained from and treated as published in Reference [17].

In mammals, multiple copies of genes encode the canonical histone proteins [11]. These genes share extensive sequence similarities and are expressed during the S phase of the cell cycle. Histone variants emerged in mammals for each family of histones, except for H4. These variants include somatic histone variants (H2A.X, H2A.Z, Macro-H2A, H2A.B, and H3.3) and testis-specific variants (TS H2A.1, H2A.L, TS H2B.1, H2B.L, subH2B, and H3T) [11]. These non-allelic variants differ little in their amino acid sequences and are highly conserved between human and mouse (Figure 1). In contrast to canonical histones, histone variants are usually present as single-copy genes that are expressed throughout the cell cycle [12,13]. They ensure specific functions and confer specific structural properties to the nucleosome. In the particular context of spermatogenesis, for example, histone variants are key players involved in the differentiation of adult germ stem cells into mature spermatozoa. In that specific case, the sequential replacement of canonical histones by histone variants facilitates chromatin compaction. Indeed, the few variations in their amino acid composition confer specific properties to these variants and are either associated to repressive or to active chromatin states by destabilizing the structure of the nucleosome. For instance, H2A.Z was largely associated to active gene expression, while macro-H2A variants were essentially found in repressive chromatin regions [14]. Moreover, some of these variants are expressed in the latest stages of spermatogenesis, suggesting their important role in the packaging of male germ cell DNA [15]. Post-translational modifications on histones can also significantly impact chromatin conformation, and thus regulate the accessibility of DNA to transcription factors. Many of these modifications can occur together to modulate gene expression. The testis-specific histone variant H2B (TS H2B.1) is the major variant form of somatic H2B. Surprisingly, *Th2b* KO has no effect on male infertility due to a compensatory mechanism mediated by the addition of methyl groups at H4R35, H4R55, H4R67, and H2BR72 [16]. These findings suggest that histone modifications as well as histone variants are “docking sites” and a network for the recruitment of protein factors to facilitate the chromatin remodeling, and thus the regulation of gene expression. However, there is still a long way to go to obtain a complete understanding of the complex interplay between histone variants, PTMs, and protein effectors.

**Figure 2:**
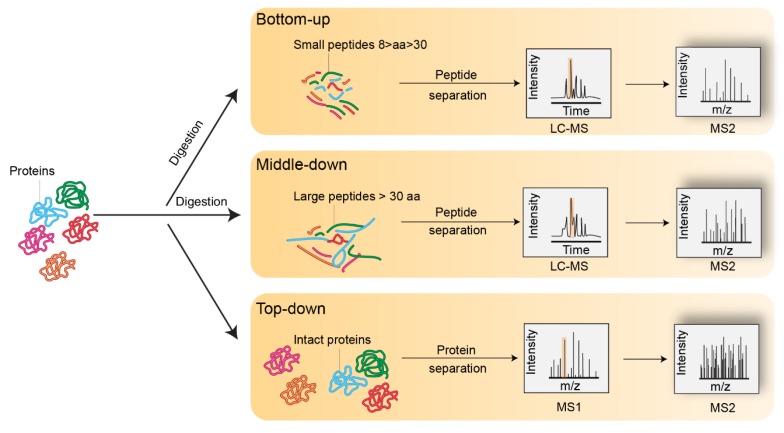
Representation of bottom-up, middle-down, and top-down mass spectrometry experiments.

A key challenge for scientists studying histones and their variants lies in deciphering the complexity of histone variants, identifying PTM combinations, and understanding their role in pathophysiological contexts. Historically, this challenge has been addressed using antibody-based methods, such as western blot, immunofluorescence analysis, and chromatin immunoprecipitation (ChIP). However, the sequence homology between histone variants and the large array of histone PTMs that exist are incompatible with the generation of highly specific antibodies. In addition, antibody-based assays require knowledge of the type and position of the modification of interest. As a result, histone antibodies are not always specific for their targets and often generate cross-reactivity [18]. Additionally, two neighboring PTMs within the same histone may cause epitope occlusion, and thus impede recognition of the target PTM. Finally, these approaches lack high-throughput capabilities, and each PTM must be analyzed separately, which makes the analysis of multiple PTMs occurring within the same histone expensive as well as time- and effort-consuming [19,20]. As an alternative, mass spectrometry (MS)-based proteomics is a valuable approach for the identification and quantification of co-occurring PTMs within a single protein sequence. Historically, three main MS-based approaches, namely bottom-up, middle-down, and top-down methods, were developed and adapted for the investigation of histone PTMs (Figure 2). All of these approaches have been extensively applied to histones and successfully mapped over 500 different PTMs [5]. The PTMs identified include a broad range of classical histone modifications such as lysine or arginine methylation (Kme/Rme), lysine acetylation (Kac), O-GlcNAcylation (S/T-GlcNAc), ubiquitination (Kub), and serine and threonine phosphorylation (Sph/Tph). The landscape of lysine modifications was significantly broadened by the recent discovery of acyl chains, such as propionylation (Kpr) and butyrylation (Kbu) [21], crotonylation (Kcr) [22], succinylation (Ksucc) [23], malonylation (Kma) [24], 2-hydroxyisobutyrylation (Khib) [25], and glutarylation (Kglu) [26]. Altogether, the large diversity of PTMs, along with their possible combinations on histones, render their MS analysis incredibly complex. Here, we discuss advances made in sample preparation and instrumental methods. Computational methods used to detect and quantify histone variants and their PTMs are also reviewed.

## 2. Extracting Histones from Biological Samples

Many protocols have been published to extract histones from mammalian cells and tissues. A landmark protocol was published by Shechter et al. [27] describing two classically used options. The procedure consists in isolating nuclei using a hypotonic lysis buffer. As histones are highly basic proteins, with an isoelectric point close to 10, they can be extracted using either acidic (classically sulfuric acid) or high-salt buffers. However, histone extraction protocols were not specifically designed for MS analysis, and caution needs to be taken for a successful comparative analysis of endogenous PTMs. First, some PTMs are sensitive to the extraction method, such as the phosphorylations on Ser and Thr residues, which are labile at very low pH [27]. Moreover, it is necessary to avoid/remove MS-incompatible reagents such as detergents and high salt concentrations. The type of lysis mechanism should also be evaluated to maximize histone yield. To investigate the histone PTM patterns between different samples, the extraction should be done as fast as possible to reduce artefactual changes mediated by residual epigenetic effectors. Finally, histone proteins can be further fractionated on an SDS-PAGE gel to remove possibly abundant high-molecular weight proteins that would impede the study of low stoichiometry PTMs; alternatively, extracted histone samples can be loaded on a C8 or C18 column to produce pure fractions of each histone subtype [28,29].

## 3. Bottom-Up Mass Spectrometry Analysis of Histones

Several MS methods have been developed for use with histones; these methods analyze different sizes of protein fragments. This review will focus on the most widely adopted bottom-up approach; middle-down and top-down approaches are reviewed elsewhere [30,31]. The bottom-up analysis offers reliable comparisons between samples, but for the identification and quantification of PTMs, subtle pitfalls from histone preparation for proteomic analysis to MS data interpretation have to be considered.

The bottom-up approach involves proteolysis of the histones into short peptides (8–30 amino acids), the sequences of which can then be determined by MS/MS fragmentation. Different proteolytic enzymes can be used, but trypsin remains the protease of choice for its efficiency and specificity of cleavage. However, because histones are very rich in lysine and arginine residues, some regions of histones get chopped into peptides which are too short for HPLC retention and successful identification by MS/MS. Garcia et al. [32] developed a protocol to chemically derivatize lysines, thus blocking trypsin cleavage. This chemical treatment adds a propionyl group to unmodified lysine residues and restricts the action of trypsin to arginine residues, mimicking an Arg-C-like proteolysis, but with improved reliability. As a result, peptides suitable for MS-based analysis are generated. However, this derivatization procedure is not compatible with the study of naturally occurring propionylation events [33], and also partially alkylates the hydroxyl groups on serine/threonine residues. In addition, the frequently observed addition of a propionyl group to methylated lysines is strictly isobaric (i.e., of exactly the same mass due to identical compositions in atoms) with butyrylation. This issue can be overcome through the use of an isotope-labeled propionic donor group, such as D5-propionic anhydride [34] or D5-butyryl anhydride [35]. Garcia et al. [33] assessed the ionization efficiency of 12 chemical derivatization groups, and propionic anhydride appeared to be one of the best agents.

In 2011, Zhao and colleagues [22] identified crotonylation as a new lysine PTM and described its role during spermatogenesis. The existence of crotonylation, like the other PTMs reported over the past decade, was validated by analyzing a synthetic peptide of the same sequence and bearing the hypothetical modification, and then verifying strict co-elution during LC separation of the synthetic and endogenous species, and their highly similar fragmentation patterns. This new PTM was searched for using four different sample preparation protocols to increase histone sequence coverage and extend the repertoire of modified sites. Histone extracts were thus (1) digested in-solution using trypsin without chemical propionylation, (2) chemically propionylated after in-solution tryptic digestion, (3) chemically propionylated before in-solution tryptic digestion, and (4) in-gel digested after SDS-PAGE gel separation. Among the four methods tested, in vitro propionylation before tryptic digestion of histones gave the highest sequence coverage for histones H1, H2A, and H2B. In-gel digestion after SDS-PAGE separation provided the best coverage for histones H3 and H4, 75–80% coverage for H2A, H2B and about 60% for H1 [22]. Thus, these protocols, with and without in vitro chemical modification of non-modified Lys residues, can provide complementary PTM identifications.

## 4. Data Interpretation

Mass spectrometry fragmentation (MS/MS) data is interpreted using dedicated search engines which compare experimental MS/MS spectra to theoretical peptide fragmentation patterns and identify the best match. A number of search engines are now publicly available on the web (summarized here https://omictools.com/database-search-category) alongside the commercial solutions. These search engines rely on a “protein sequence database” which is explored to identify proteins and their PTMs from the experimentally acquired data. These databases should be comprehensive, non-redundant, and well annotated to allow optimal MS/MS data interpretation. Two major repositories exist, hosted by the National Center for Biotechnology Information (NCBI: http://www.ncbi.nlm.nih.gov/) and the Universal Protein Resource (UniProt: http://www.uniprot.org/). However, the current versions of these public databases contain many redundant entries (UniProtKB or NCBI). The NCBInr or UniRef100 databases contain only non-redundant proteins, but their histone nomenclature does not follow the current community standards [36]. Recently, a proteomics-oriented and manually curated resource of mouse and human sequences, named MS_histoneDB, was released [11]. Each histone entry in this database is annotated in accordance with the current nomenclature and unified with the “HistoneDB2.0” database [36,37]. This resource is provided in a format that can be directly read by MS/MS data interpretation programs. Our team used MS_histoneDB to interpret proteomics data for histones extracted from mouse testis. Several histone variants that had so far only been inferred by homology or detected at the RNA level, were detected by MS analysis, thus confirming their existence at the protein level [11].

Another critical parameter to specify before launching the interpretation of MS/MS data for histone peptides is the list of expected modifications. Close to 20 different structures of PTMs have thus far been described on histones [38]. However, they cannot all be considered simultaneously by most search engines, which only accommodate a limited number of variable PTMs (e.g., a maximum of nine for Mascot). The performance of eight programs was compared with respect to their capacity to identify PTMs on histone peptides, and pFind and Mascot appeared to yield good performances [39]. As an alternative to matching experimental MS/MS spectra to theoretical spectra deduced from known protein sequences, programs designed to perform de novo sequencing of MS/MS data have been successfully used for sequences containing few modifiable residues. For peptide sequences such as the N-terminal peptide of H4 GKGGKGLGKGGAKR, however, the several variable PTMs on the four lysines make the search space far too broad to obtain satisfactory results. Finally, a few groups have developed software tools to identify mass shifts associated with as-yet undescribed PTMs. PTMap is one such tool based on a sequence alignment algorithm; it selects sequence-information-rich MS peaks, infers precise localization of PTM sites, and assigns a score based on spectrum quality [40]. PTMap was successfully used to discover propionylation and butyrylation on histones [41].

## 5. The Puzzle of Histone PTMs

**Figure 3:**
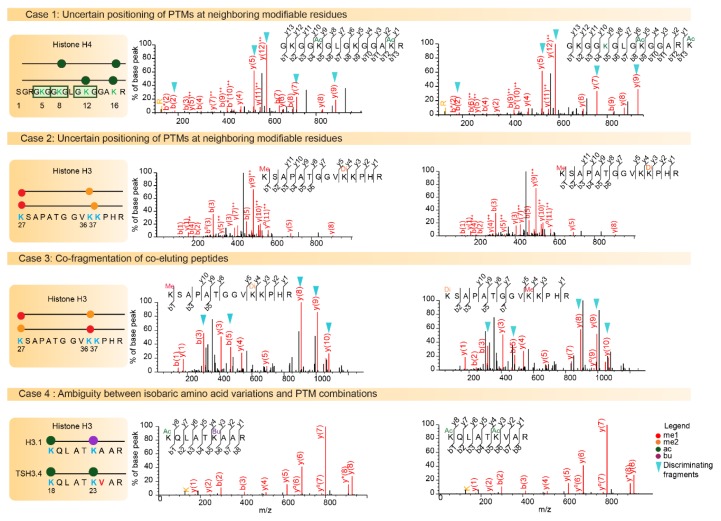
Challenges of histone post-translational modifications (PTM) assignments. The Mass Spectrometry fragmentation (MS/MS) spectra of higher energy collisional dissociation (HCD) fragmented histone peptides are shown. Case 1 illustrates positional isomers for the di-acetylated N-terminal tail of histone H4 containing the four Lysine residues K5, K8, K12, and K16. The left spectrum illustrates the identification of the first positional isomer indicated in the diagram, with a Mascot identification score of 65. On the right, the same spectrum is interpreted with fragments of the second possible modified sequence matched with a score of 56. Fragment ions b2, y5, y7, and y9 and y12++ can be used to assign acetylation on different lysine residues. Case 2 illustrates the difficulty associated with confidently assigning a Lys36 or Lys37 di-methylation to peptide *K_27_SAPSTGGVK_36_K_37_PHR*, as it relies only on the weak-intensity y4 fragment to discriminate between these two modifiable residues. Case 3 shows the probable co-fragmentation of the co-eluting peptides K_27_me1-K_36_me2 and K_27_me2-K_36_me1. The two positional isomers are matched to this MS/MS spectrum with Mascot scores 31 and 29, respectively. Discriminating b3, b5, y8, y9 and y10 fragment ions are detectable at high intensity in the spectrum. Case 4 illustrates ambiguity between amino acid variation and combination of PTMs. The masses of peptides K_18_QLATK_23_acVAR from TS H3.4 and K_18_QLATK_23_buAAR from H3.1/H3.2 are strictly equal. A diagnostic ion at *m*/*z* 126.091 indicates the presence of an acetylated Lys residue in the fragmented peptide, and thus orients toward the identification of acetylated TS H3.4.

Over the past ten years, >500 individual histone PTMs have been mapped by MS analysis [5]. The complexity of investigating PTMs within histone proteolytic peptides is due to: (i) the frequent existence of strictly isobaric peptides, (ii) the uncertain positioning of PTMs on neighboring modifiable residues, (iii) the difficulty of distinguishing between several isobaric PTM combinations, (iv) the potential masking of isobaric histone amino acid variations by PTM combinations (Figure 3). Finally, the above aspects not only complicate identification of the correct sequence and of the PTM sites, they also make it very difficult to quantify the relative abundance of positional isomers that often co-elute, at least partially.

### 5.1. Frequent Existence of Strictly Isobaric Peptides

First, at the protein sequence level, some histone variants generate strictly isobaric peptides that can only differ by their amino acid order. For example, the mammalian H2A.L.1 isoforms share very high similarity. Those encoded by the *H2al1j* and *H2al1a* genes differ by a swap of an amino acid triplet: peptides GEFPLSLVDR from H2A.L.1 (*H2al1j*) and GELPFSLVDR from H2A.L.1 (*H2al1a*) differ by the swap of FPL residues to LPF. They co-elute using a classical elution gradient on a C18 column, and can only be qualitatively and quantitatively distinguished by targeted proteomics (Selected Reaction Monitoring or Parallel Reaction Monitoring) using discriminating fragment ions, such as y6 and y7 ions [17]. Second, a given amino acid sequence containing several modifiable residues (such as Lys and Arg) and bearing a set of PTMs can exist as multiple positional isomers (i.e., same modification occurring on different residues of a given peptide sequence), which complicates the identification and relative quantification of these diverse forms, as detailed below.

### 5.2. Uncertain Positioning of PTMs on Neighboring Modifiable Residues

LC-MS/MS analysis of the N-terminal fragment of histone H4 can identify acetyl modification across four lysine sites (K5, K12, K8 and K16) [42]. In this case, it is difficult to confidently assign di- or tri-acetylation to specific lysine residues, because positional isomers frequently co-elute, and as a result co-fragment. Identification results produced by the Mascot search engine on this sequence (Figure 3, case 1) demonstrate that several possible sequences only differing by their PTM positions were suggested with similar identification scores. Feller et al. [43] developed an optimized protocol to elucidate the problem of PTM positioning within a given peptide. They used chemical acetylation of all modified and mono-methylated lysines with deuterated acetic anhydride, which adds a 3-Da mass shift and distinguishes endogenous from chemical acetylation. The acetic derivatization gives the originally unmodified peptides the same chemical properties as the endogenously acetylated peptides to make comparison possible.

### 5.3. Ambiguity between Several Isobaric PTM Combinations

H3 residues K27 and K36 have been described as harboring numerous combinations of acetylation/methylation states (ranging from me1 to me3 on either H3K27 or H3K36) [44]. In fact for a precursor of the same mass, peptide K_27_SAPSTGGVK_36_K_37_PHR may in theory be modified as follows: K_27_me1-K_36_me2, K_27_me1-K_36_me1-K_37_me1 and K_27_me1-K_37_me2. The absence of efficient HCD fragmentation between residues K36 and K37 makes PTM positioning very difficult, if not impossible (Figure 3, case 2). Moreover, K_27_me1-K_36_me2 and K_27_me2-K_36_me1 co-elute, making quantification of these two species difficult when using a classical exploratory approach. In such cases, the positional isomers must be quantified by considering specific fragments of each peptide form, which consists of implementing a targeted proteomic analysis. Indeed, these two positional isomers can be distinguished by monitoring fragment ions y8 to y10 (see details in Figure 3 case 3).

**Figure 4:**
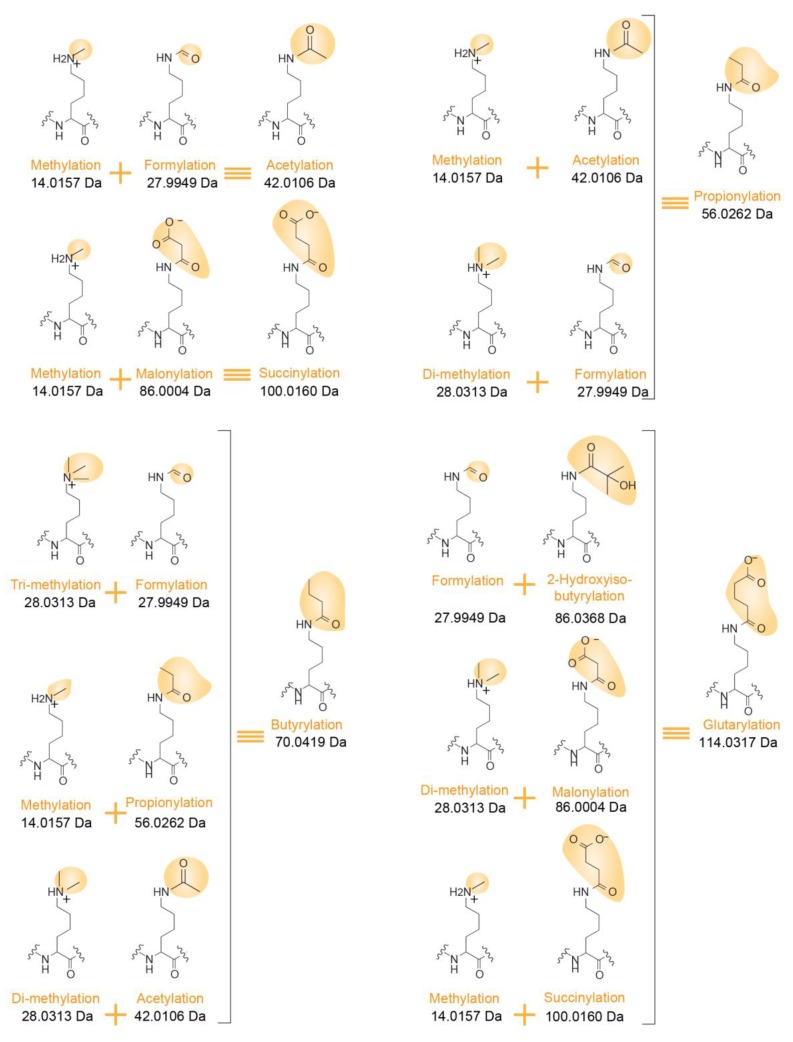
Examples of isobaric PTM combinations. Differentiating structures in the Lys side chain are highlighted in yellow with an indication of their corresponding masses.

Confidently assigning PTMs to specific histone residues is made even more challenging as multiple PTM combinations can correspond to the very same mass increment. For instance, as already mentioned above, the mass of butyrylation equals the sum of the masses of methylation and propionylation. Other possible correspondences are indicated in Figure 4. Notably, both PTMs occurring in vivo and chemical modifications that may be added during protein/peptide sample processing can affect the peptide sequences submitted to LC-MS/MS analysis. Formylation and acetylation can exist on the side chain of Lys residues, but can also modify the N-termini of peptides when formic acid and acetic acid are used during in-gel digestion and LC separation, for example. Confidently assigning a PTM to the Lys residue present at the N-terminal end of a peptide (such as K_27_SAPSTGGVK_36_) can thus be uncertain.

### 5.4. Distinguishing between Isobaric Amino Acid Variations and PTM Combinations

**Figure 5:**
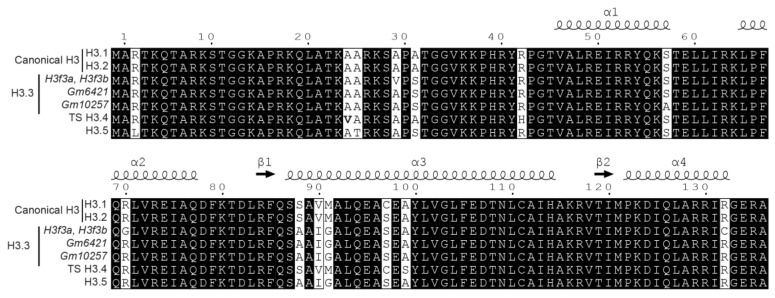
Sequence alignment for mouse histone H3 and its variants.

Canonical histones and their variants are highly similar and can differ by a small number of amino acid variations (Figure 1). In some cases, the mass shift induced by the amino acid change between histone variants equals the difference of mass between two PTMs. Thus, the combination of amino acid variations and PTMs can generate isobaric peptides, making the confident assignment of PTMs even more complicated. For example, the H3 family includes two canonical histones, H3.1/H3.2, and three variants, H3.3/H3.5/TS H3.4. These proteins are highly similar, as shown in Figure 1 and Figure 5. TS H3.4 differs from canonical H3.1/2 by two amino acid residues, namely A24V and R42H. H3.1K23 has been described as acetylated in mouse studies [45]. The combination of acetylation and of a valine residue produces a peptide with the same mass as a butyrylation and an alanine residue. Hence, incomplete fragmentation makes it difficult, if not impossible, to distinguish between the TS H3.4 variant bearing an acetylation within the K_23_VAR sequence and canonical H3 bearing a butyrylation in the K_23_AAR region (Figure 3, case 4). Interestingly, modified amino acids can generate immonium ions, which, after neutral losses, yield unique diagnostic ions. These ions can be used to detect the presence of a modification within peptides. Closer examination of the MS/MS spectrum in case 4 from Figure 3 revealed the presence of an ion characteristic of acetylated lysine at *m*/*z* 126.091, whereas no signal was detectable at *m*/*z* 154.123, which would have indicated the presence of a butyrylated Lys residue in the fragmented peptide. This observation validated the presence of an acetylated Lys in the analyzed peptide, and thus identified the sequence as K_18_QLATK_23_acVAR from TS H3.4.

Scrutinizing these diagnostic ions is also very useful for the delicate case of peptides which have a Lys residue in their N-terminal position. Indeed, they can discriminate between a peptide with an N-terminal lysine bearing an acetylation on its side chain, and a peptide combining a methylation on the lysine side chain and a formylation at its N-terminus as a result of using formic acid during sample preparation and analysis. Several diagnostic ions have been reported for most of the commonly investigated PTMs (Table 1). The production of immonium and diagnostic ions can be improved by using the fragmentation mode Higher energy Collisional Dissociation (HCD) rather than Collision-Induced Dissociation (CID) [46]. In addition, whereas CID spectra often lack these fragments due to the instability of lower-mass species in the ion trap, HCD spectra include them with a high mass accuracy. The large numbers of CID spectra acquired in recent years on instruments such as the hybrid LTQ-Orbitrap instruments actually provide ambiguous identifications due to the strictly isobaric nature of several PTM combinations. More frequent use of HCD fragmentation now should eliminate ambiguities by focusing on diagnostic ions. The SPIID program (SPectral Immonium Ion Detection) was recently developed to facilitate the mapping of known diagnostic ions through the binning of high-resolution MS/MS spectra, and may facilitate this task [47].

## 6. Positional Isomer Quantification

Co-eluting positional isomers cannot be quantified by classical bottom-up data-dependent acquisition (DDA) because their respective signals merge at the MS1 level. To determine the relative abundance of co-eluting isobaric peptides, targeted approaches like SRM (Selected Reaction Monitoring) or PRM (Parallel Reaction Monitoring) must be implemented. Recently, Wei Li and colleagues [63] developed an SRM approach to quantify combinatorial PTMs born by histone H3 K_27_ and K_36_. They successfully applied their method to investigate the distribution of modified H3K_27_-K_36_ in mouse organs. Other studies also successfully used SRM and PRM to assess the level of histone H3 acetylation in human brain tissue from patients with advanced Alzheimer’s disease or to quantify low-abundance histone modifications [64,65]. Jaffe et al. [66] conducted a large-scale analysis of histone modifications and implemented PRM to quantify histone H3 modifications from 115 cell lines contained in the Cancer Cell Line Encyclopedia.

A few software tools have been developed to address the challenging process of quantifying differentially modified isobaric peptide forms. Garcia et al. [67] developed the EpiProfile program to distinguish positional isomers, using discriminating fragment ions in their MS/MS spectra and extracting the chromatographic peak area under the curve based on information available on peptide retention times. The software successfully differentiated between H3 K9/K14 mono-acetylated peptides; H3K27me1 *versus* H3K36me1 peptides; and H4 K5/K8/K12/K16 mono-acetylated or di-acetylated peptides.

Data-Independent Acquisition (DIA) has been effectively used to investigate histone PTMs [68,69,70], offering the combined benefits of SRM/PRM and DDA analysis modes. The DIA approach fragments all precursor ions within a given retention time and *m*/*z* window. As a result, DIA is unlikely to produce missing values, and thus provides higher quantitative reproducibility. Moreover, DIA can differentiate between isobaric and co-eluting peptides as data analysis and quantitation can be performed from both MS1 and MS2 scans. For instance, it can resolve and quantify isobaric H3 peptides such as the sequence K_18_QLATK_23_AAR acetylated on either K18 or K23 [68]. DIA was successfully used to profile 62 PTMs in histones H3 and H4 and to assess dynamic changes in methylation and acetylation in response to exposure to HDAC inhibitors [70]. In addition, Sidoli et al. [71] used DIA to differentially quantify isobaric phosphopeptides. It should be noted, however, that the application of this method is still limited due to the difficulty represented by the combination of several species in the same MS/MS spectrum, the sensitivity required to detect unique fragment ions of the same type for the two isobaric isoforms, and limitations of the computational tool when calculating the relative ratio between more than two isoforms. The increasing interest in DIA should lead to the development of software for data processing which can identify and quantify isobaric peptide modifications.

## 7. Conclusions

Over the past decades, MS-based proteomics workflows have been established and refined for the investigation of a broad range of histone PTMs. The significant improvements to computational tools have provided new insight into developmental biology, through the identification of histone PTM crosstalk, but further developments are still needed. The discovery of an array of new PTMs (e.g., acylations) and the need to consider subtle amino acid variations among histone sequences, especially in specialized tissues such as the testis, still make the deciphering of histone PTM patterns an exquisitely complex game. Being able to comprehensively and reliably identify and quantify these combinations will pave the way for clinical epigenetics, through profiling of histone modifications for diagnostic and prognostic purposes.

## Figures and Tables

**Table 1:** Diagnostic ions useful for PTM assignment. Immonium ions (IM); side-chain fragmentations (SC); neutral losses (NL).

Amino Acid Residues	PTMs	Diagnostic Ion Type	*m*/*z*	MS Fragmentation	References
Lysine	Acetylation	IM-NH_3_	126.091	CID, HCD	[48,49,50,51]
	Methylation	IM-NH_3_	98.0964	CID, HCD	[52,53]
	Dimethylation	IM	112.1	CID, HCD	[52,53,54]
	Trimethylation	NL	59.0735	CID	[51,52,53,54]
	Propionyl	IM	140.106	CID	[45]
	Crotonyl	__	__	__	__
	Butyryl	IM	154.123	HCD	[45]
	Malonyl	__	__	__	__
	2-hydroxyisobutyryl	__	__	__	__
	Succinyl	__	__	__	__
	Gluratyl	__	__	__	__
	Formyl	IM	112.0756	HCD	[47,51,55]
Arginine	Methylation	NL	73.064	ETD	[52,53,54,56,57,58]
	Methylation	NL	31.0422	ETD	[52,53,54,56,57,58]
	Dimethylation	SC	46.0651	CID	[59,60]
	Dimethylation-symmetric/asymmetric	SC	71.0604	CID	[59,60]
	Dimethyl-symmetric	NL	31.0417	CID	[56,57,61]
	Dimethylation-symmetric	NL	46.0651	CID	59,60,64]
	Dimethylation-asymmetric	NL	45.0573	CID	[56,57,61]
Tyrosine	phosphoryl	NL	216.0426	CID	[47,62]
